# A rare case: Endobronchial solitary mixed papilloma

**DOI:** 10.5578/tt.20239712

**Published:** 2023-09-22

**Authors:** B. Demirkol, M.A. Karagöl, E.G. Uğur Chousein, Ö. Adıyeke, N. Büyükpınarbaşılı, A. Bahadır, S. Yurt, M.A. Özgül

**Affiliations:** 1 Clinic of Pulmonology, University of Health Sciences Başakşehir Çam and Sakura City Hospital, İstanbul, Türkiye; 2 Clinic of Pulmonology, University of Health Sciences, Yedikule Chest Diseases and Thoracic Surgery Training and Research Hospital, İstanbul, Türkiye; 3 Clinic of Anesthesia and Reanimation, University of Health Sciences Başakşehir Çam and Sakura City Hospital, İstanbul, Türkiye; 4 Clinic of Pathology, University of Health Sciences Başakşehir Çam and Sakura City Hospital, İstanbul, Türkiye

**Keywords:** lung cancer, endobronchial, papilloma

## Abstract

**ABSTRACT:**

**The evolution of endobronchial ultrasound usage in modern era:**

Endobronchial solitary papillomas are extremely rare lung neoplasms originating from the bronchial surface epithelium. They often present with cough or recurrent hemoptysis. These tumors are benign, but they should be followed closely because they may even have a low probability of malignant transformation features. It should be kept in mind that malignancy may develop especially if the patient is a smoker. Although the etiology is not known for certain, it is thought to be caused by human papillomavirus in some cases. A 43-year-old male patient was admitted with a complaint of chronic cough. Rigid bronchoscopy was performed for diagnostic and therapeutic purposes after imaging techniques revealed a lesion obstructing the lumen of the right main bronchus. The pathology result was reported as mixed bronchial papilloma. We aimed to present our case because of its rarity and to indicate that chronic cough must be further evaluated.

## Abstract (tr)

**Ender bir vaka: Endobronşiyal soliter mikst papillom:**

Endobronşiyal soliter papillomlar, bronşiyal yüzey epitelinden köken alan oldukça nadir akciğer tümörleridir. Sıklıkla öksürük ve tekrarlayan hemoptizi ile kendini gösterir. Bu tümörler iyi huylu tümörlerdir ancak malign transformasyon özelliği gösterme olasılıkları düşükte olsa olabileceği için yakından takip edilmelidir. Özellikle hasta sigara içiyorsa malignite gelişebileceği akılda tutulmalıdır. Etiyolojisi kesin olarak bilinmemekle birlikte bazı olgularda human papilloma virüsünün neden olduğu düşünülmektedir. Kırk üç yaşında erkek hasta kronik öksürük şikayeti ile başvurdu. Görüntülemede sağ ana bronş lümenini tıkayan lezyon saptanması üzerine tanı ve tedavi amaçlı rijit bronkoskopi yapıldı. Patoloji sonucu mikst bronşiyal papillom olarak rapor edildi. Nadir görülmesi ve kronik öksürüğün ileri değerlendirme gerektirdiğini belirtmek amaçlı olgumuzu sunmayı amaçladık.

## INTRODUCTION

Endobronchial solitary papillomas are rare lung papillary tumors of epithelial origin. Histologically, they have three subtype groups: squamous cell papilloma, glandular papilloma, and mixed papilloma ( 1 ). They are considered premalignant lesions although the possibility of malignant transformation is rare ( 2 ). A rare case, histopathologically diagnosed as endobronchial mixed papilloma with endobronchial treatment through the rigid bronchoscope, is presented.

## CASE REPORT

**Figure 1:**
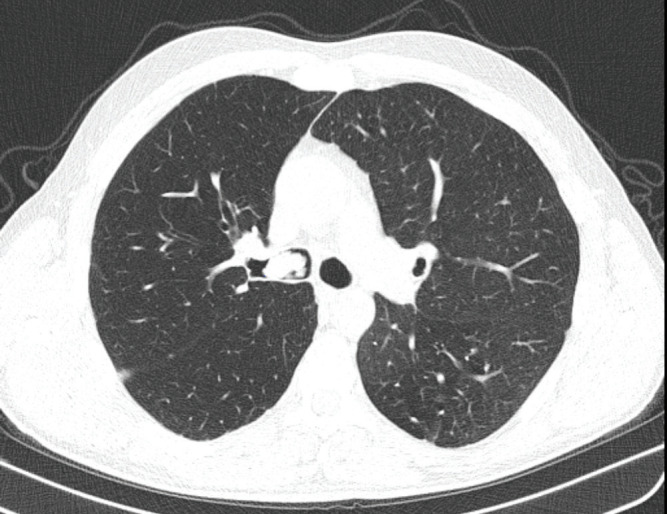
Soft tissue density obliterating the lumen of the right main bronchus on thoracic CT.

**Figure 2:**
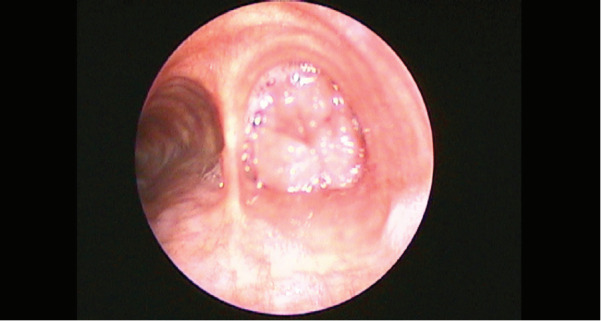
Endobronchial lesion substantially obliterating the lumen of the right main bronchus.

**Figure 3:**
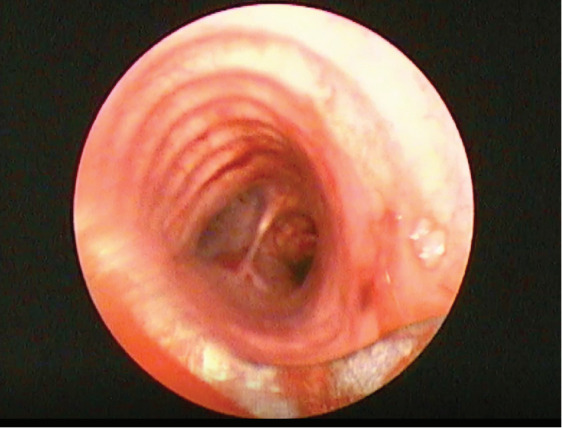
Almost complete patency of the right main bronchus was achieved with the procedure.

**Figure 4:**
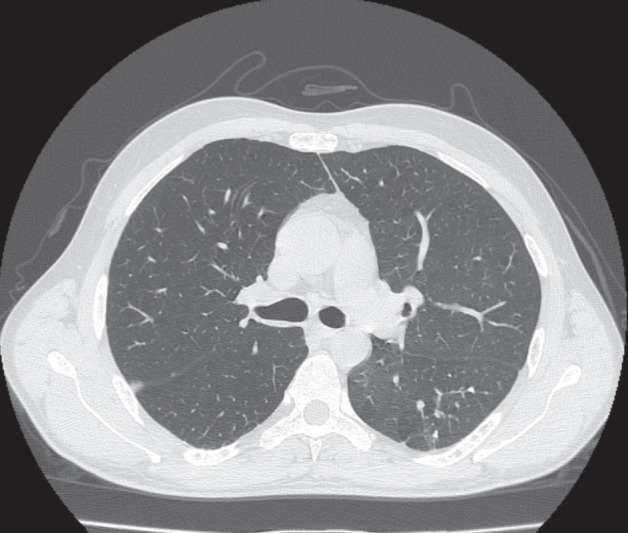
The right main bronchus lumen is seen to be patent following the interventional bronchoscopic procedure on thorax CT.

A 43-year-old male was admitted to the hospital with a complaint of chronic cough for about two years. An endobronchial lesion was detected in preliminary radiological examinations and an endobronchial treatment following further examinations was planned as an inpatient. He had no diagnosis of a known disease. He was an active smoker with a history of 20 packs/year. Physical examination revealed normal bilateral respiratory sounds. In room air, the oxygen saturation level was 96%, and the pulse rate was 87/ min. All blood examination tests were in normal ranges. Posteroanterior chest X-ray was within normal limits. Thoracic computerized tomography (CT) showed a 20 x 13 mm endobronchial lesion obliterating the lumen of the right main bronchus ( Figure 1 ). A lobulated soft tissue lesion defined in the lumen of the right main bronchus with minimally increased FDG metabolism was reported in positron emission tomography-computed tomography (PET)-CT. Rigid bronchoscopy revealed a cauliflower-like polypoid lesion originating from the lateral wall of the right main bronchus ( Figure 2 ). The peduncle of the lesion was coagulated with argon plasma coagulation (APC). The lesion was separated from the wall by mechanical resection which was done by the rigid tube itself, and cryotherapy was applied to the remaining tissues ( Figure 3 ). Optimal airway patency was achieved in the thorax CT performed after the procedure ( Figure 4 ).

**Figure 5:**
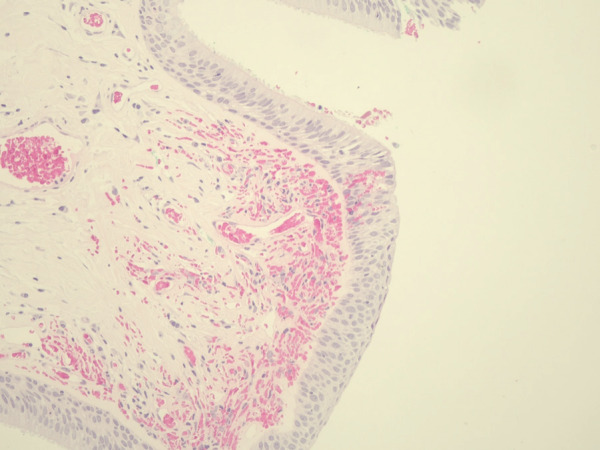
Ciliated and squamous epithelial transition on the polyp surface.

**Figure 6:**
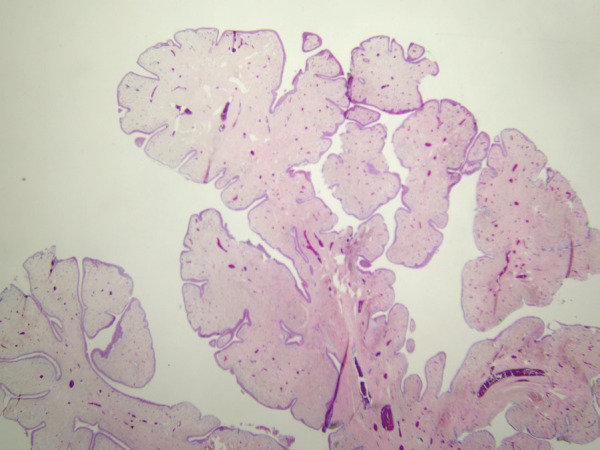
Presence of papillary projections in the polyp.

Pathological examination revealed a papillary polypoid lesion with fibrotic and vascularized stroma and ciliated columnar and squamous metaplastic epithelium and was reported as mixed bronchial papilloma ( Figure 5 , Figure 6 ).

## DISCUSSION

Pulmonary papillomas are rare benign tumors of the lung and have been reported as case reports in the literature. They are more common in men, may show malignant transformation, and smoking is an important risk factor ( 3 , 4 ).

When the literature was reviewed, it was seen that the majority of cases, which is 39 in total, were reported in Japan between 1975 and 2004. The ages of cases ranged from 22 and 80 years and 30 of them were males ( 2 ). Considering that most of the patients were males, our case fitted this sex distribution.

Patients are usually asymptomatic. If symptomatic, they most commonly present with hemoptysis, chronic cough, or obstructive pneumonia ( 5 ). Chest radiography may be within normal limits, which may cause a delay in diagnosis. The most common images seen on thoracic CT are polypoid lesions narrowing the endobronchial lumen. Atelectasis and bronchiectasis are other common accompanying radiological findings. This present case was diagnosed as bronchial papilloma by bronchoscopic biopsy of a soft tissue density compatible with an endobronchial lesion obstructing the bronchus lumen detected on Thoracic CT of a male smoker who was presented with chronic cough.

Papillomas originate from squamous epithelial cells. Histopathologically, they are divided into three subtypes; squamous, glandular, and mixed type ( 6 ). Squamous cell papillomas are the most common papillomas and there are case reports in the literature ( 7 ). Among these cases, cases of squamous papilloma type with malignant transformation have been reported ( 3 ). Mixed and glandular papillomas are also seen less frequently and are reported as single case reports in the literature ( 8 ). This case with a histopathology of mixed type of papilloma is extremely rare.

Papillomas may be centrally or peripherally located according to their localization. Centrally located papillomas may present with endobronchial presentation. Endobronchial ones are mostly seen in the lobar and segmental bronchi but rarely seen in the main bronchi. In our case, the endobronchial lesion was observed in the right main bronchus.

Patient-specific treatment options are planned for each individual patient. Segmentectomy is performed in parenchymal cases where the size of the papilloma is smaller and limited, and bronchoscopic methods are used in cases with endobronchial lesions ( 2 ). Bronchoscopic methods include photodynamic therapy, yttrium aluminum garnet (YAG) laser vaporization, and snare electrocautery. It has also been reported that surgical methods should be prioritized in cases of incomplete resection due to recurrence. Surgical resection methods are used in cases where the lesion size is larger or deeply invaded. In our case, bronchoscopic resection was performed due to the endobronchial localization of the polypoid lesion. The fact that he was a smoker and the large volume of the lesion raised suspicion of pre-malignancy, so a close follow-up strategy was planned for him. Radiological and bronchoscopic evaluations showed no recurrence in the first-year follow-up of the patient.

Solitary bronchial papilloma should be kept in mind in the differential diagnosis of male smokers admitted to the hospital with chronic cough or hemoptysis after the exclusion of other common causes. Patients who underwent bronchoscopic treatment should be followed up closely because of the possibility of recurrence and the potential for malignant transformation.

## CONFLICT of INTEREST

The authors have no conflict of interest to declare.

## AUTHORSHIP CONTRIBUTIONS

Concept/Design: BD, MAK, MAÖ, SY, AB

Analysis/Interpretation: BD, MAK, ÖA, EGUC

Data acqusition: MAK, ÖA, NB

Writing: BD, MAK, MAÖ, SY, AB

Clinical Revision: BD, MAK

Final Approval: BD
